# An Overview of the nMPRA and nHSE Microarchitectures for Real-Time Applications

**DOI:** 10.3390/s21134500

**Published:** 2021-06-30

**Authors:** Vasile Gheorghiță Găitan, Ionel Zagan

**Affiliations:** 1 Faculty of Electrical Engineering and Computer Science, Stefan cel Mare University, 720229 Suceava, Romania; 2 Integrated Center for Research, Development and Innovation in Advanced Materials, Nanotechnologies, and Distributed Systems for Fabrication and Control (MANSiD), Stefan cel Mare University, 720229 Suceava, Romania

**Keywords:** nMPRA architecture, hardware RTOS, rapid reaction to stimuli, fast context switch, resource multiplication

## Abstract

In the context of real-time control systems, it has become possible to obtain temporal resolutions of microseconds due to the development of embedded systems and the Internet of Things (IoT), the optimization of the use of processor hardware, and the improvement of architectures and real-time operating systems (RTOSs). All of these factors, together with current technological developments, have led to efficient central processing unit (CPU) time usage, guaranteeing both the predictability of thread execution and the satisfaction of the timing constraints required by real-time systems (RTSs). This is mainly due to time sharing in embedded RTSs and the pseudo-parallel execution of tasks in single-processor and multi-processor systems. The non-deterministic behavior triggered by asynchronous external interrupts and events in general is due to the fact that, for most commercial RTOSs, the execution of the same instruction ends in a variable number of cycles, primarily due to hazards. The software implementation of RTOS-specific mechanisms may lead to significant delays that can affect deadline requirements for some RTSs. The main objective of this paper was the design and deployment of innovative solutions to improve the performance of RTOSs by implementing their functions in hardware. The obtained architectures are intended to provide feasible scheduling, even if the total CPU utilization is close to the maximum limit. The contributions made by the authors will be followed by the validation of a high-performing microarchitecture, which is expected to allow a thread context switching time and event response time of only one clock cycle each. The main purpose of the research presented in this paper is to improve these factors of RTSs, as well as the implementation of the hardware structure used for the static and dynamic scheduling of tasks, for RTOS mechanisms specific to resource sharing and intertask communication.

## 1. Introduction

In general, an operating system (OS) is responsible for managing the hardware resources of a computing system and the applications it hosts. A real-time operating system (RTOS) implements many similar functionalities (especially those for high end embedded devices), but is especially designed to run applications with a very precise synchronization and evolution over time and with a high degree of reliability and security. Usually, this behavior is important for automation systems where a program delay could cause a security hazard (for hard real-time systems (RTSs)) and also downtime can be costly. In order for an OS to be considered real-time, it must have a maximum time (e.g., worst case execution time—WCET) for each critical operation it supports (implements). Among these operations we mention are scheduling and dispatching and OS functions calls. To be considered “real-time”, an OS must have a known maximum time for each of the critical operations it performs (or at least be able to guarantee that maximum most of the time). Some of these operations include OS calls and interrupt management. As a classification, OSs that can guarantee an absolute maximum time for these operations are commonly referred to as “hard real-time”, while operating systems that can only guarantee a maximum of most of the time are called “soft real-time”. In practice, each RTOS solution specifies different performance characteristics, and as a result, the user must carefully investigate these characteristics and test whether they are suitable for the needs of his application, especially since the determination of WCET times depends on how the application is organized. Airbag and video streaming applications are extreme examples of hard real-time and soft real-time. For the first, a small timing error can make the difference between life and death, and for the second, the sporadic loss of frames can be unnoticed, i.e., it has no devastating consequences. An important issue is that of correctly programming an application in the context of an RTOS. Proper programming can guarantee the execution of a software with a temporary evolution and a consistent synchronization. In this regard, RTOS offers programmers a high degree of prioritization of tasks and provides mechanisms to verify compliance with important deadlines. From the implementation point of view, we can classify RTSs as without RTOS; with RTOS fully implemented in software or implemented in software/hardware (usually hardware accelerators are used for different typical RTOS components, either externally usually in a field programmable gate array (FPGA) or internally as coprocessors or separate hardware blocks); or with RTOS implemented in hardware, HW-RTOS (minimally implements the scheduler and dispatcher, resource sharing mechanisms, synchronization mechanisms, communication mechanisms between threads, and interrupt management). A natural question is: when do we use one of these implementations? The choice is dictated primarily by the application’s requirements for latency (response time), jitter, compliance with deadlines and their criticality (hard real-time—zero degrees of tolerance, firm real-time—unacceptable quality reduction, or soft real-time—accept quality reduction and this reduction is acceptable), the ability to easily calculate WCET, solving priority inversions, the need to use or not a rich set of application programming interface (API) functions (productivity in programming), the degree to which the application is oriented toward intensive computing up to input/output operations, etc.

To feel the gap from software to hardware implementation, consider the following example. Let us see how to handle a signal event in an RTOS software and in a HW-RTOS (HW_nMPRA_RTOS in this case) when the signal is sent to a higher priority thread.

For RTOS software: If we are working, for example, with Keil RTX5, we call an RTOS API function of type retValue = osThreadFlagsSet (tid_mbe1ThreadCycle, FLAG_TIMER4_SLOT), where tid_mbe1ThreadCycle is the thread identifier and FLAG_TIMER4_SLOT is the name of the flag. Some action: tests to see where the event setting comes from (Interrupt Service Routine (ISR) or thread); tests the parameters and the state of the object and if there are no errors; sets the flag with an atomic operation; records the processing of the object and returning from the interrupt (if applicable); test if the thread for which the flag was set is waiting for this event, and if so, this thread becomes higher priority than the thread that called the function or was preempted by the interrupt that called the function; saves the current state of the called or aborted thread; and restores the state of the thread that received the flag because it was waiting for it and launches it. As it is complicated, it requires atomic operations and many instructions.

What is happening in HW-RTOS (HW_nMPRA_RTOS): An osThread–FlagSet like function must send a signal or an interrupt software event. We analyze the first solution. When a thread wants to activate a signal, a 32-bit value with the Signal bit 1L is written in the grSSMR0 (Signal Synchronization and Message Register File) register and will receive a 1-bit signal value from the grSSMR0 register or a 0-bit signal value that represents a failure (most likely indicating no free SSMR, see Section 4.4). If the operation is successful, in the next machine cycle after the write operation the destination thread has the signal and message event set to 1L. If the destination thread was waiting for this signal and is higher than the thread that sent the signal, then in the next machine cycle, the wait Rj instruction ends and the program counter (PC) loads with the start address of the handler to manage this event. Everything is done in one to two cycles depending on the position of the event relative to the rising clock of the clock (about one cycle if it is very close to the rising edge of the clock, about two cycles if the rising edge of the clock has just passed). Depending on the implementation, one or two additional machine cycles may occur. This example illustrates the advantage of hardware implementation. Of course, the price of this advantage is paid by the cost of hardware complexity. Additionally, in-depth research has been performed on the processors used in RTS and RTOS architectures, and the results indicate that some or all of the components need to be incorporated into the hardware due to its ability to increase parallel processing and, therefore, to reduce the response time of embedded systems. Results in this regard have been published in [1,2]. The latency introduced by RTOS implemented in the software is given by intensive switching of thread contexts (also depends on the type of applications), API execution time that depends on dispatching, generating periods with interrupts disabled (e.g., when scheduling in supervisor mode, critical regions, etc.), the time required to handle queues (insertion, removing, sorting, and determining the highest priority) that are closely related to typical OS structures such as the Task Control Block, number of queues, and OS clock management (tick). In addition, due to the variable number of accesses that generate overhead, a jitter is created and consequently a WCET that is difficult to define. HW-RTOS by parallel processing can be improved in the sense of reducing the time given by the latency generated by RTOS.

There are situations in which the rapid response of a sensor must be accompanied by the following path: sensor ⇒ microcontroller ⇒ actuator, just as fast and often deterministic (with a WCET that can be determined or measured). In such situations, the overhead given by software RTOS is one of the reasons why a fast (or optimal) response is more difficult to obtain and less deterministic. The response of the software running on the microcontrollers must deal with signals from different sensor types and less internal mechanisms (hardware/software) in order to guarantee the best signals processing time.

One of the current trends in RTS is the migration to increasingly complex processor architectures with more predictable execution and isolation of thread contexts, thereby achieving safer and more efficient applications. Due to the complexity of industrial and automotive applications and response times, problems such as “motor will not move smoothly”, “control precision is poor”, and “network performance is slow” occur when using RTOS implemented in software [1]; consequently, it has been timely to design and validate RTOS hardware functionality in highly compute-intensive embedded systems, thus enabling more efficient CPU time management.

Programmable logic technology based on a FPGA is a fundamental component of a lot of hardware design and can be extremely useful in microprocessor architecture research. Affordable FPGA circuits with a high number of logical gates [3] are used as hardware support for the implementation and testing of proposed concepts [4]. The real-time aspects of some RTSs are critical in fields such as aviation, automotive, robotics, and movement command and control. For these systems, predictability is a very important feature. The ability to develop RTOS hardware accelerators, hardware schedulers, or HW-RTOS alongside RTOSs will allow rapid response times (low latencies) to external stimuli and events, i.e., asynchronous events, and controllable deterministic behavior in both simulations and practical demonstration deployment using FPGA technology [5]. The objectives of the innovative solutions described in this paper are to design and validate a high-performance nMPRA (multi pipeline register architecture, where *n* is the degree of multiplication) and nHSE (hardware scheduler engine for *n* threads) microarchitectures by implementing RTOS-specific functions in hardware, minimizing the kernel latency. In this context, the nMPRA concept and nHSE module provide an innovative solution with a kernel latency to events of one or two processor cycles, which is a significant improvement over the software solutions of RTOSs or software/hardware hybrid implementations. nMPRA is a custom architecture with multiple (*n*) pipeline registers. The purpose of this project was to design, implement, test, and validate an innovative concept named HW_nMPRA_RTOS (a unified acronym for nMPRA, nHSE, and RTOS API), which uses and extends concepts regarding the multiplication of CPU data path resources, the nHSE as scheduling module, and the hardware implementation of an RTOS.

Why do we consider that HW_nMPRA_RTOS includes an HW-RTOS? The part called hardware RTOS ensures priority scheduling. Static scheduling algorithms, such as Monotonic Rate and Round Robin, can be easily implemented, but in the future dynamic scheduling, algorithms such as Earliest Deadline First (EDF) [1], etc., can also be implemented. This is possible because the priority of a hardware instance of a thread (instPi) is programmable; there are two (interchangeable) priority correspondence tables—thread identifier. The hardware instance for thread 0 (instP0) has access to all architecture resources and can write the unused table with a new set of priorities after which it can switch it to active. The wait Rj instruction (or an equivalent instruction depending on the architecture chosen for implementation) can synchronize the thread with seven events (time, deadline 1, 2, watchdog timer (WDT), mutex, signal and message, and interrupt event), which allows implementation using a single instruction (after a preconfiguration) to obtain time-type functions, to gain access to critical resources by automatically acquiring a mutex, or to synchronize or communicate with other threads using signal and messages or interrupt events. All these are basic facilities for simple programming of a real-time application for a low embedded device without additional software. When we refer to the low embedded device, we have in mind the category from the ARM (Advanced RISC Machines) classification called Cortex-Mx. With several C inline functions that include some instructions in the assembler, specific RTOS software functions can be obtained. The notion of RTOS refers in this paper to the kernel. A networking stack and many I/O functionalities are usually provided as middleware (see Keil ARM) or as separate libraries. Implementing them in hardware could have an unjustifiable cost.

In this paper, it has been considered that the description of RTOS mechanisms helps the real-time application programmer to correctly understand the hardware facilities available to them to get a real-time response to some events generated by sensors, and be able to make correspondence easier with APIs provided by software implemented RTOS. For example, the implementation of hardware RTOS has a unique and fast execution time to search in lists (it does not depend on the position in the list, changing the position in a list, etc., because everything is done in parallel for all items in the list).

The contributions made by the authors in this paper consist of presenting original methods to reduce latency for events handled by HW_nMPRA_RTOS, reducing the jitter effect by designating a unified space of priorities for tasks and events to be handled by the nHSE, and augmenting the processor execution level. The specific derivative contributions of this research project consist in System on Chip (SoC) implementation of nMPRA and nHSE at the level of MIPS32 coprocessor 2 (COP2), the scheduler registers being explained in detail in the specifications of the nMPRA processor. The HW_nMPRA_RTOS concept together the practical results presented in this paper have been designed using the MIPS32 Release 1 ISA. Microprocessor without Interlocked Pipelined Stages (MIPS) provides the user with a system of coprocessors for extending the functionality of the basic CPU. Coprocessor 2 is available to the user. The degree of novelty and relevance of the proposed architecture is demonstrated by the results published in the prestigious IEEE TVLSI [6] and Electronics [7] journals.

After a brief introduction, Section 2 analyzes other similar projects published in the literature. Section 3 is dedicated to the proposed CPU architecture, and Section 4 describes the hardware scheduler concept and validates the results of the HW_nMPRA_RTOS implementation. Section 5 focuses on discussions regarding the integration of the CPU using FPGA resources and compares similar architectures, and Section 6 describes the programming paradigms for nMPRA. Section 7 presents the final conclusions.

## 2. Related Work

The nMPRA concept appeared for the first time in [8], which defined it as custom-designed Multi Pipeline Register Architecture. The remarkability of the architecture is reflected in the name itself by specifying that the pipelined registers are multiplied, which allows the hardware context of the thread being executed to be saved, making it easier to stop an instance of the pipeline at any time and to change the context in a single clock cycle. The multiplication of hardware resources is (partly) illustrated in [7].

A solution with general purpose register (GPR) multiplication and resources dedicated to each thread is provided in [9], for example, and in the block diagram of the precision-timed (PRET) microarchitecture [10]. The multiplication of pipeline registers is applied in [11], although it is simple and multiplies only the instruction fetch (IF) and instruction decode (ID) pipeline registers. Returning to [8], although MPRA is not clearly defined, the HSE module is described. As a novelty, a unified space of priorities is proposed for both interrupts and threads, which allows some threads to be prioritized over interrupts. Special instructions are proposed for HSE programming, which may create inconvenience when compiling (these must allow for the expansion of ISA (instruction set architecture)). The scheduling of threads can be based on RM (rate monotonic) or EDD (earliest due date) algorithms with programmable task priorities. A single timer is proposed for all threads, but in this case, comparators and adders are needed, which complicates the hardware. The solution of using an autoreload counter for each thread using a common clock is simpler and represents a feasible option. In [12], the authors proposed organizing the GPR in the form of several banks of general registers for general use, but it was subsequently not possible to demonstrate the usefulness of this extension, which was conceived as a local stack that would not require the repetition of push and pop in the stack memory. In [6], the HSE was fully redefined, and a more elaborate solution was tested and subsequently validated. The term MPRA has been replaced by nMPRA, where *n* is the number of hardware support instances for the execution of *n* software execution threads, and HSE has been replaced by nHSE because it will be scheduled for *n* execution threads that will be run on *n* instances of nMPRA. Only one nMPRA hardware instance is active at any given time. An instance of nMPRA execution contains logical combinational blocks of the five pipeline stages (IF, ID, EXECUTE (EX), DATA MEMORY (MEM), and WRITE BACK (WB)), which are common resources shared by all nMPRA instances, as well as those from the private hardware resources associated with the software thread that is being instantiated (GPR, pipeline, and PC; status and control register file; flag file; work registers and/or counters [7]). A more detailed description of the architecture can be found in [6] with the appropriate terms that represent the architecture at that time.

Real-time embedded systems are those systems that provide a correct answer within a predetermined time interval. In critical real-time applications, obtaining a correct response after the deadline is insufficient and can no longer be taken into account [13]. In [14], the authors propose a CPU implementation based on the RV32IM ISA five-stage pipeline recommended for hard RTSs. The performances of the uRV Core project are oriented towards determinism of execution over performance, having a minimum set of control and status registers (CSR), the performances being guaranteed with Coremark 1.0 benchmark. The PULP (parallel ultra-low power) platform was developed to explore new and efficient architectures for ultra-low-power processing [15]. The main objective of this project was to develop an open and scalable research platform. The specific objectives of the project were to increase energy efficiency as well as to meet the computing requirements of IoT applications that require flexible processing of data streams generated by multiple sensors, such as accelerometers and low-resolution cameras. For this purpose, the authors proposed an innovative microcontroller and a multi-core platform characterized by outstanding low energy consumption and large-scale adaptable performance. The Merasa project [16] was developed to obtain a processor architecture that can be successfully used for hard RTSs. The main feature of this project is task execution predictability for a simultaneous multithreading technique, in which both hard real-time (HRT) and non-real-time (NHRT) threads are executed at the same time. The XMOS processor presented by May in [17] has a scalable and flexible architecture. The pipeline execution uses the entire central processing unit, even if the number of active execution threads is less than four. The proposed kernel used in Meakin [18] was designed to implement the entire MIPS instruction set. Other than XUM-specific extensions, MIPS specifications are strictly implemented. Since XUM involves the study of multi-core processing, at the end of implementation in the FPGA chip, the authors obtained several CPU cores and an interconnection network available on a parallel architecture.

Research carried out in the field of microprocessor architecture proposes concepts that integrate both RTOSs functions and the task scheduler, which are fully or partially implemented in hardware. Thus, scheduling algorithms as well as inter-task synchronization and communication mechanisms are integrated into the hardware, guaranteeing a typical RTSs response time. In these projects, the software still has the task of switching the thread contexts. This paper proposes the implementation in hardware of nHSE and RTOS mechanisms, as well as the switching of contexts based on thread resources multiplication. These achievements have the advantage of minimal time to handle events attached to the processor instances, leading to low power consumption and overall performance.

## 3. nMPRA and nHSE Overview

**Figure 1:**
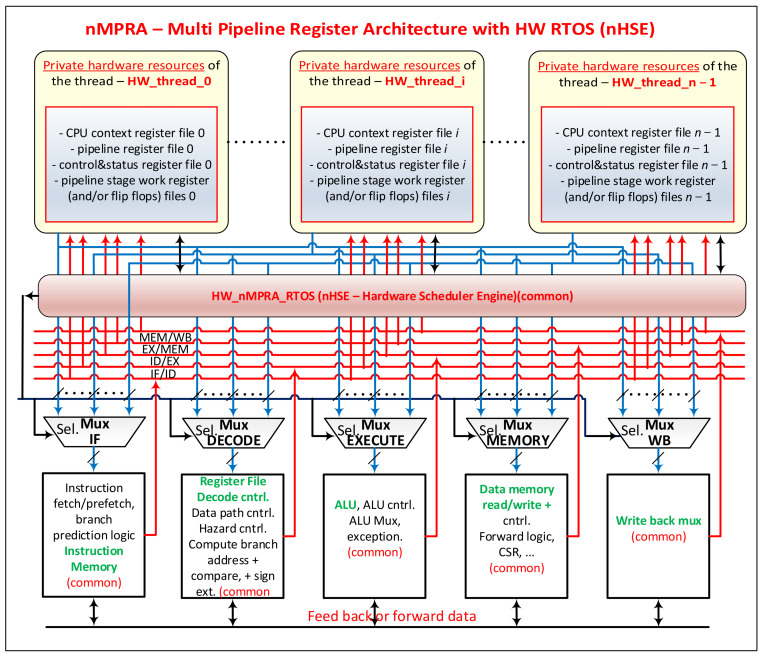
Arrangement and notations for the nMPRA + nHSE concept.

**Figure 2:**
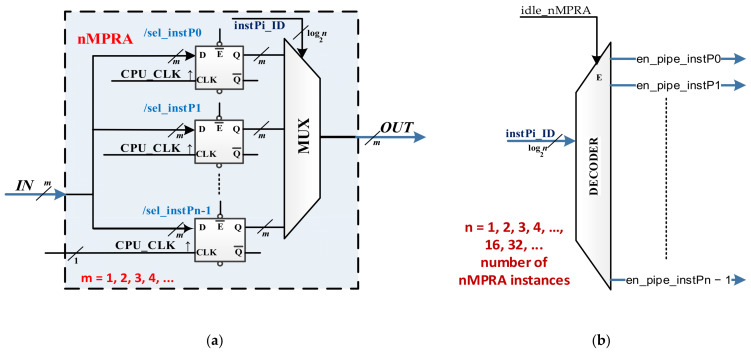
Multiplexing the multiplied memory elements: (**a**) multiplication of storage resources (flip-flops, work registers, counters, etc.); (**b**) nMPRA instance decoding based on instPi_ID bits.

With the expansion of nMPRA architecture and facilities, in this paper, we redefine the system based on the architecture illustrated in Figure 1. The private resources of threads, referred to as HW_thread_i with *i* = 0, …, *n* − 1, are as follows:

- GPR (for MIPS32 and RISC-V, there are 32 general-purpose 32-bit registers, for ARM 16 general 32-bit registers, etc., to which it is possible to add other registers available to the programmer, such as the current program status register (CPSR) for ARM, PC for RISC-V, Hi and LO multiplication registers for MIPS32, etc.);
- Pipeline registers (for example, MIPS32 includes the IF/ID, ID/EX, EX/MEM, and MEM/WB pipeline registers);
- Status and control registers (e.g., RISC-V CSR, ARM control register, RTOS-associated registers implemented through hardware, etc.);
- Condition and status indicators, work registers, and counters. In the design stages, which are essentially dependent on the chosen BASELINE (BL_NAME) (which may be an implementation of a MIPS32, Cortex-Mx, RISC-V, PowerPC, etc.), the presence of flip-flops, work registers, and counters in the shared common area (HW_COMMON_CPU) that are not saved in pipeline registers must be replicated at the level of each HW_thread_i (see Figure 2, where instPi is the static identifier of the nMPRA instance, and en_pipe_instPi is the resource selection signal for instance *i*). All of these files depend on the BL, the type of architecture (MIPS32, Cortex-Mx, RISC-V, PowerPC, etc.) and the concrete implementation (HW_nMPRA_RTOS can be implemented in the form of a coprocessor, e.g., MIPS32 (COP2); RISC-V CSR; etc.).

The hardware that has the common attributes in Figure 1 are as follows:

- The resources needed to deploy RTOS-specific components in hardware (HW_nMPRA_RTOS);
- Multiplexers (or factory switches) for coupling the hardware resources of a thread with the pipeline stages (with a major influence on the working speed of the nMPRA execution instance, power consumption, FPGA clock distribution, and resource utilization);
- The common logical part of the pipeline stages (IF, ID, EX, MEM, and WB) for a five-stage pipeline architecture.

The architecture can comprise 2–8 pipeline stages and 2–32 threads or even more if necessary (we do not recommend this). Figure 2a presents a resource multiplication solution with multiplied memory elements. All entries are common, and the memory bits have the same clock signal, but at some point, only one set is active because the flip-flop validation entries are connected to the output of a decoder (only one output is active at a time if the decoder is validated, i.e., the *E* entry is active, which means that there is at least one nMPRA instance running), as shown in Figure 2b.

If the *E* validation entry is inactive, all instances are IDLE, and none of them are active. From a hardware perspective, an nMPRA instance is a complete processor, which we denote by instPi with *i* = 0, …, *n* − 1, implemented on a specific BL (with a certain ISA architecture) and a specific implementation of it. A single thread runs on an nMPRA instance, i.e., the instance whose private resources are embedded in the hardware instance. At some point, nMPRA executes a single instance. We can then write the following:

(1) $$\mathrm{instPi}\;=\;\mathrm{HW}\_\mathrm{thread}\_i\;+\;\mathrm{BL}\_\mathrm{name}\;\left(\mathrm{PIPELINE}\_\mathrm{STAGE}\right)+\;\mathrm{HW}\_\mathrm{nMPRA}\_\mathrm{RTOS}\left(\mathrm{TYPE}\right)+\;\mathrm{MUX}\;\left(\mathrm{PIPELINE}\_\mathrm{STAGE}\right)\;$$

where the components are defined as follows:

instPi: Instance *i* for nMPRA;

HW_thread_i: Private hardware resources for thread *i*;

BL_name (PIPELINE_STAGE): Name of the baseline with the number of pipeline stages used;

HW_nMPRA_RTOS (TYPE): RTOS implemented in hardware for *n* threads, where TYPE = coprocessor, block that uses CSR, standalone, etc.;

MUX: Resource multiplexers shared by threads, dependent mainly on the number of pipeline stages.

The BL used for implementation in this study is the XUM project (R1) described in [19] with sources in [20], which is a MIPS32 processor; HW_nMPRA_RTOS uses coprocessor 2 with a five-stage pipeline. There is another implementation that uses the RISC-V architecture called uRV with sources in [21] and a four-stage pipeline. The CSR block was the design basis for HW_nMPRA_RTOS. For these architectures, the nMPRA instances can be written as follows:

(2) $$\mathrm{inst}\_\mathrm{Pi}\;=\;\mathrm{HW}\_\mathrm{thread}\_i\;+\;\mathrm{BL}\_\mathrm{MIPS}32v5\_\mathrm{XUM}\left(5\right)+\;\mathrm{HW}\_\mathrm{nMPRA}\_\mathrm{RTOS}\left(\mathrm{COP}2\right)+\;\mathrm{MUX}\left(5\right)$$

(3) $$\mathrm{inst}\_\mathrm{Pi}\;=\;\mathrm{HW}\_\mathrm{thread}\_i\;+\;\mathrm{BL}\_\mathrm{RISCV}32\mathrm{IM}\_\mathrm{uRV}\left(4\right)+\;\mathrm{HW}\_\mathrm{nMPRA}\_\mathrm{RTOS}\left(\mathrm{CSR}\right)+\;\mathrm{MUX}\left(4\right)$$

In the nMPRA specifications, the following prefixes are defined:

- Control registers (cr_), which contain specific instPi with thread-level access;
- Local registers (lr_), which are part of the private address space of each instPi;
- Global registers (gr_), which are part of the global nMPRA address area and can be accessed by all instPi;
- Monitoring registers (mr_), which can be accessed either locally or globally.

Other rules are mentioned in the description of each register. Control and monitoring registers are usually local registers. Global registers are always prefixed with gr_, regardless of their role (status or control). All nMPRA instances are identical, except instP0, which has certain additional facilities: for example, it is the active instance after RESET, it has access to some of the other instances’ registers, and it always has an execution priority of 0, which is the highest priority in the system. Other facilities are specified in this paper when appropriate.

## 4. Real-Time Operating System Implementation in nMPRA

The RTOS implementation in nMPRA, called HW_nMPRA_RTOS, also includes the nHSE described in [6]. HW_nMPRA_RTOS performs the basic functions of an RTOS implemented in hardware with exceptional reaction times that range from a machine cycle to less than three machine cycles (in rare cases), depending on the situation. Even for a processor operating at 20 MHz, delays can range from 50 to 150 ns, i.e., a very fast response. Increasing the frequency, e.g., to 100 MHz, translates the time range to 10–30 ns (this is the delay without execution time of before and after instructions.) For microcontrollers operating at 200 MHz frequencies that use a specialized and optimized RTOS software, the thread-switching time is at least a few microseconds if there is hardware support at the microarchitecture level for quick thread switching.

For HW_nMPRA_RTOS, the following functional requirements are defined:

- The nHSE defined in [6] is implemented (which contains event generation logic, a static scheduler, and a dynamic scheduler support);
- Manage:
  ○ Access to shared resources using mutexes;
  ○ Synchronization and communication between threads using signals/message events;
  ○ External interrupts (managed as events);
  ○ Control and monitoring registers.
- Monitor:
  ○ Time events;
  ○ The running and idle times of each instPi and BL_nMPRA as a whole;
  ○ The identifier of the instPi that is running (instPi_ID).
- It is optional to implement a dynamic scheduling algorithm (e.g., Round Robin).

**Figure 3:**
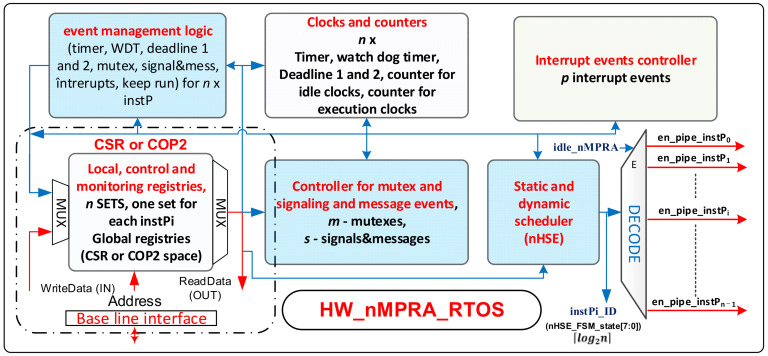
Block diagram for HW_nMPRA_RTOS, which also includes the nHSE for BL_nMPRA implemented with RISC-V or MIPS32v5.

Figure 3 shows the block diagram for HW_nMPRA_RTOS. It comprises the following functional modules:

- Static and dynamic scheduler (nHSE);
- Event management logic;
- Interrupt event controller (for *p* interrupt-type events);
- Controller for mutex, signals, and message events (for *m* mutexes and *s* signals/messages);
- Clocks and counters;
- Local, control, and monitoring registers;
- Baseline interface (BL_MIPS32v5 or BL_RISCV32IM).

These modules are described in the following subsections.

### 4.1. Event Management Logic

**Figure 4:**
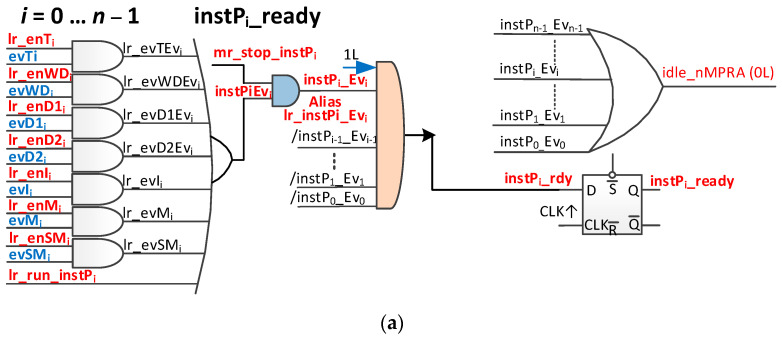

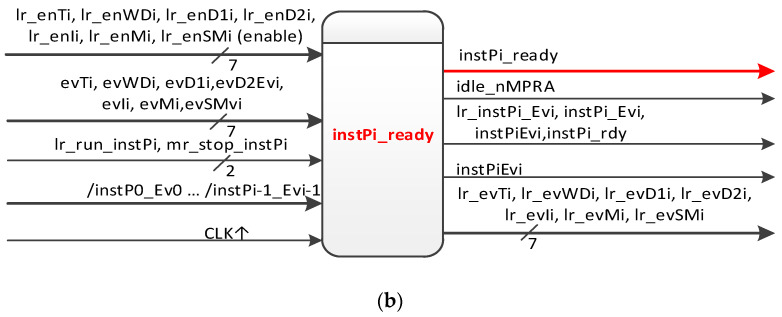
Event management logic for instPi. (**a**) Logical scheme; (**b**) block diagram.

The operation of the system shown in Figure 4a (where the “/” sign indicates a bitwise complement value) is as follows. If one or more of the seven mentioned events occurs and at least one is validated, then the instilEvi signal is activated. If the mr_stop_instPi signal is activated (only nMPRA instP0 can enable/disable it), then the instPi_Evi signal is activated. If no higher priority nMPRA instance is active (/instP0_Ev0, …, /instPi − 1_Evi − 1), the signal instPi_rdy that is connected to the data entry of the D flip-flop is activated and will be stored by the first positive edge of the nMPRA clock, activating the instPi_ready signal, which, in turn, is part of the logic that generates the instPi_ID that is ready for execution and has the highest static priority (lowest number).

The clearing of the event may result in the nMPRA processor losing control because, in the next clock cycle, the signal instPi_ready deactivates and stops the respective instPi. As a result, if the instPi execution is to continue, then the lr_run_instPi signal must be activated before the event is reset.

It can then be disabled automatically or by soft writing 0 to the lr_run_instPi bit (see crTRi register). The block scheme is shown in Figure 4b. The functional block presented in Figure 4 is intended to manage the input events originally presented in [6], which are defined as follows:

- Global, which can be used by any instPi:
  ○ Interrupt events—evIi_j;
  ○ Events generated by mutexes—evMi_k;
  ○ Events generated by synchronization and message sending (signals/messages)—evSMi_l or, briefly, signals;
- Local, specific to each individual instance instPi:
  ○ Event generated by WDT—evWDTi;
  ○ Event generated by the timer (tick)—evTi;
  ○ Event generated by deadline 1—evD1i;
  ○ Event generated by exceeding deadline 2—evD2i.

where

*i* = 0, …, *n* − 1, *n* = maximum number of instPi of nMPRA);
*j* = 0, …, *e* − 1, *e* = maximum number of interrupt events;
*k* = 0, …, *m* − 1, *m* = maximum number of mutexes; and
*l* = 0, …, *s* − 1, *s* = maximum number of signals.

For FPGA deployment, there is a restriction that specifies that three-state circuits can only be used at the level of the external pins of the circuit. As a result, the original scheme in [6] must be adapted. In this respect, we discuss the signal instPi_ready. These signals can be used as alias signals for en_pipe_instPi (see Figure 3). The instPi_ready signal is only active among those instPi instances that are ready to run at that time, and it has the highest priority in the system (lowest identifier). At this point, instPi_ID must be created as $\log_{2}n$ based on instPi_ready signals (see Figure 4.b). Another problem is that, when no nMPRA instances are ready to run, the signal en_pipe_instPi (see Figure 3) must be inactive. To do this, the situation must be noted when all instPi instances are not attached to an active event or are not self-executable. This is the IDLE state of the nMPRA finite-state machine (FSM), so no instances are active, and no threads are running.

This paper proposes an improved variant of the scheme that is presented in Figure 4a by including an additional signal that detects the state in which no nMPRA instance is ready for execution, namely, the signal idle_nMPRA (0L = IDLE). As a result of the introduction of this signal, the identifier of instPi has *n* + 1 bits as the highest bit to indicate whether (i.e., 0) or not (i.e., 1) an instPi is active. The block diagram of the module that manages the ready-to-run status of an nMPRA instance (logical event management block in Figure 3) is shown in Figure 4b.

Next, the registers necessary for the implementation of HW_nMPRA_RTOS, including local, control, and monitoring registers (Figure 3), are presented. Thus, for the proper functioning of the nHSE, the following registers must be defined:

crTRi is the thread register (cr_), which is the event validation register (Table 1) that allows the event to generate (or not) the signal instPi_ready to the first positive edge of the clock (a local register that can also be read by instP0). It is a register that serves to validate (1) or inhibit (0) an event. After the CPU is reset, all bits are 0 (except the bit lr_run_instPi, which is 1L, for *i* = 1, …, *n*).

crEVi is the event register (cr_), which indicates the activated (1) or not (0) of an event (Table 2). After the CPU is reset, all bits are 0L (except for the bit lr_run_instPi, which is 1L).

cr0MSTOP is the master stop register; this is a master register for stopping instPi execution, and it is valid only for instP0 (Table 3). After the CPU is reset, all bits are 0L. The occupation of the register depends on the number of instPi.

Switching from one instance to another occurs, as mentioned above, very quickly (e.g., one or two nMPRA cycles). Synchronization with one of the desired events can be performed using the generic assembly instructions wait Rj or wait Rj, events. If there are events already pending and validated by the Rj register, the wait instruction does not block the nMPRA instance that is running it. When the wait instruction is completed, the Rj register contains pending events that are validated by the wait instruction. If this is not the situation and the bit lr_run_instPi is 0L, then the wait Rj blocks the nMPRA instance on which it is running. Upon returning from the wait instruction, the Rj register stores pending events that have been validated by the wait instruction via the Rj register (see Table 1).

**Figure 5:**
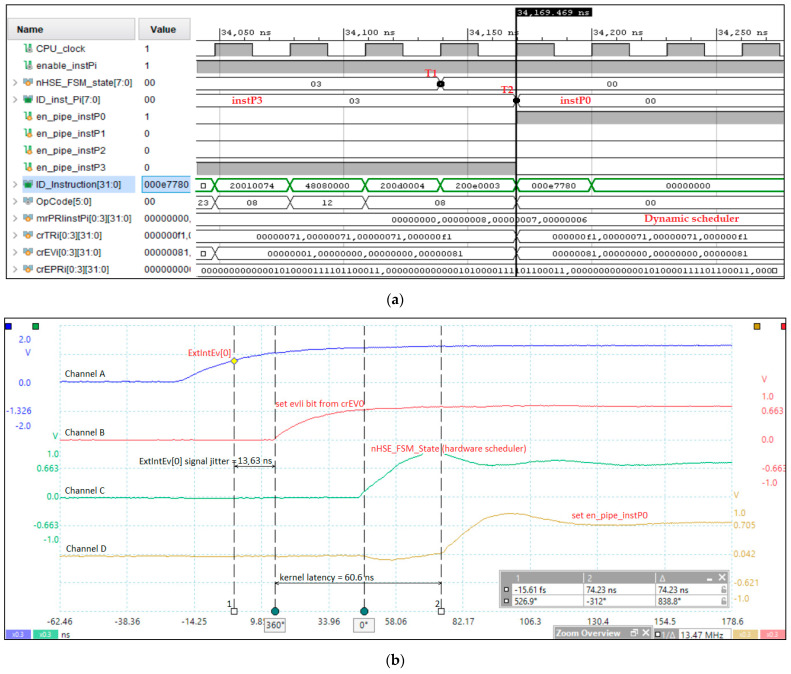
Kernel latency following project simulation and implementation based on FPGA design methodology. **(a)** Simulation related to HW_nMPRA_RTOS FSM, crTRi, and crEVi registers and assignment of instPi priorities. (**b**) Trigger the evIi event = 13.63 ns (Channel B), changing the FSM state = 43.93 ns—from ExtIntEv[0] (Channel A) to nHSE_FSM_State (Channel C) and context switch to instP0 = 74.23 ns—from ExtIntEv[0] to set en_pipe_instP0 (Channel D).

Figure 5 shows the tests performed to measure the kernel latency in the case of an event assigned to instP0, which is the highest priority event according to crEPRi (the register for prioritizing events at the instPi level, corresponding to crEVi). Based on FPGA design methodology and optimization techniques, Figure 5a illustrates the post-synthesis simulation from the design and debugging stage of the HW_nMPRA_RTOS concept at a working frequency of 33 MHz. Based on CPU harvard architecture, the dual-port on-chip memory was designed with IP Block Memory Generator 8.4 and clocked by IP Clocking Wizard 5.2. The simulator can also view the nHSE registers proposed in this paper. The integrated simulator Vivado 2018.2 Design Suite (Xilinx, Inc., San Jose, CA, USA) was used to test the data path and nHSE registers, and the project was routed, placed, and validated in the FPGA using the Virtex-7 development kit. At the T1–T2 time period, the context switching between instP3 and instP0 is performed, the latter being higher priority based on the nHSE mrPRIinstPi register, each instPi having its own hardware context that does not need to be saved. Figure 5b illustrates the test performed for the practical measurement of the kernel latency corresponding to the nHSE scheduler, i.e., the change in the output of the FSM states that generates the next transition through the nHSE_FSM_state[7:0] signals (Figure 3). Thus, tests were run to confirm that the hardware scheduler has a jitter of one clock cycle plus 13.63 ns, the time needed to trigger the evIi event (ExtIntEv[0] external interrupt captured by Channel A), but it can be any of the events (evTi, enWDi, evD1i, evD2i, evMi, or evSMi) specified in Table 2. Thus, the latency of the thread context switch in one clock cycle was simulated and tested, where the Channel D signal measures the transition of the signal en_pipe_instP0. The practical implementation of HW_RTOS_nMPRA in the FPGA validates the simulation, so the kernel latency for handling an event of the type evIi is only 74.23 ns (the trigger time of the signal ExtIntEv[0] plus the two clock cycles needed for the hardware scheduler and the thread context switch).

The kernel latency value of 60.6 ns is computed from when the evIi event is active to the moment when HW_nMPRA_RTOS fires to the first instruction of the handler routine. In [22,23], these design and testing situations are addressed, and several solutions are proposed. In [23], a solution is presented for an extreme case in which all seven types of events are validated and already pending. In the first example, the priority is set by software, and all seven indicator bits are tested sequentially. The priority is chosen by the software programmer, and the event with the lowest priority may have significant delays in its processing. A faster solution involves associating an event number, on the basis of which an offset is calculated, in a table with jumps (or calls) to program sequences for the event handler. Additionally, in [23], a hardware solution is proposed that uses a priority encoder for interrupt events, and the number of interrupts with the highest priority is saved in a register whose output is decoded, with the output of the decoder selecting a register that contains the event handler address. This event handler routine at the end must activate the lr_run_instPi bit in the crTRi register (to keep the instance in a ready-to-run state), delete the bit corresponding to the active event in the crEVi, and transfer the control back to the main loop of the thread on the corresponding HW_nMPRA_RTOS instPi. The method does not require stack saves/restores (which may exist, depending on the application). When calling the wait Rj instruction (or the equivalent of the instruction according to the BL), the current context is not considered to be important. In [22], the authors proposed prioritizing the seven events, and a similar direct transfer to the event handler routine is presented in [23].

Regardless of how control is transferred, the priority rule is the same as that for an interrupt controller (nested vector interrupt controller (NVIC), such as ARM): an event at the same priority level or lower cannot suspend the current event handler. Thus, the response to events can also be delayed by event handlers that are already running on the instance to which it is assigned. Even on the same instance of nMPRA, there is a global prioritization of interrupt, mutex, and signal/message events. The solution in [22] also proposes a register called crEPRi for selecting priorities for the seven types of events, and it has three bits for each type of event. These solutions are convenient because they allow quick access to the handler so that it can treat the event as quickly as possible. However, a more practical solution that moves trap cells from additional registers to program memory is proposed later in this paper, because, for a thorough understanding, mechanisms for implementing interrupts, mutex, and signal/message events should be discussed.

### 4.2. Static and Dynamic Scheduler

**Figure 6:**
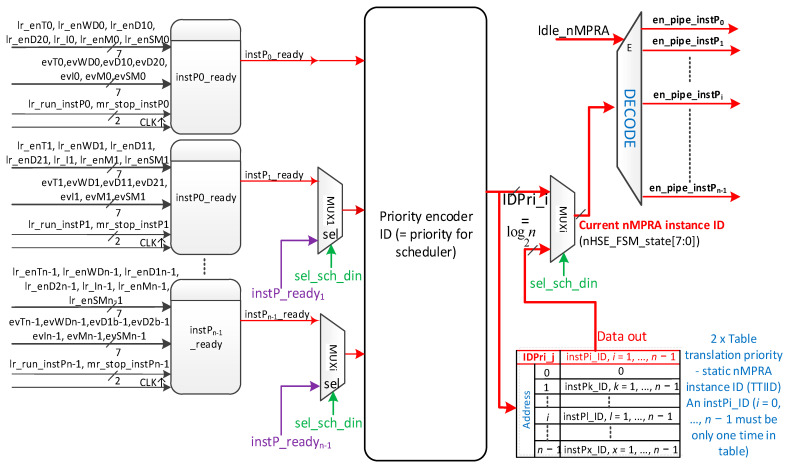
The nMPRA hardware scheduler engine for *n* instP (nHSE).

The implementation of the hardware scheduler engine, illustrated in Figure 6, is simple for static scheduling; as long as the signal scheme instPi_ready, *i* = 0, …, *n* − 1 is as presented in Figure 4. When sel_sched_dyn is 0, the priority encoder generates the binary code for the highest priority instPi. For static scheduling, the static instPi_ID is equal to the identifier of the static priority IDPri_i = instPi_ID (*i* = 0, …, *n −* 1, where 0 is the highest priority, and *n* − 1 is the lowest priority). This code, multiplexed with instPi_ID for dynamic scheduling, is decoded to generate the en_pipe_instPi selection signal of resources in the HW_thread_i module for the activation of the nMPRA instance (see the DECODE module in Figure 3 and Figure 6). Table 4 shows the truth table for an example with eight nMPRA instances, resulting in logical equations for idsPri3, idPri2, idPri1, and idPri0. The schema in Figure 6 provides support when sel_sched_dyn = 1L for dynamic scheduling in the sense that each nMPRA instance may receive another priority.

**Figure 7:**
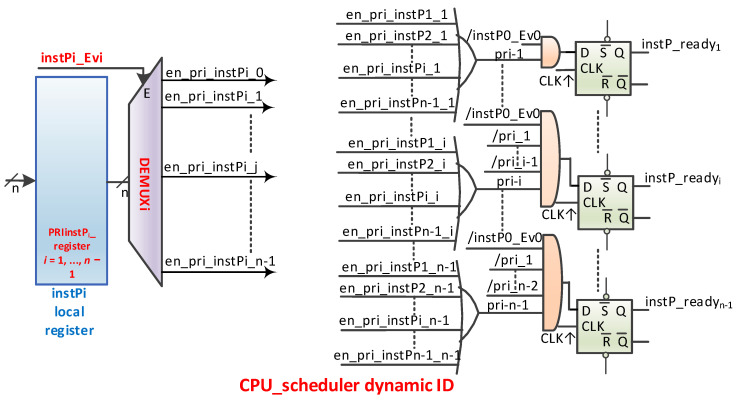
Dynamic scheduling support scheme.

For this purpose, memory (or a set of registers, which can also be double buffered for a synchronous update) is provided to translate IDPri_j with *j* = 1, …, *n* to any instPi_ID with *i* = 1, …, *n*, except that IDPri_0 is always equal to instP0_ID. To do this, it is necessary to add the hardware support shown in Figure 7, which generates the instP_readyi signals in Figure 6. The dynamic scheduler, implemented either in software and executed on nMPRA’s instP0 or in hardware, must manage the Translation Table for the Interface of Identifiers, abbreviated TTIID.

The priority of instPi_ID has values from 0 to *n* − 1, where 0 denotes the highest priority identifier and *n* − 1 denotes the lowest priority identifier. The TTIID correlates the instPi_ID whose priority has been defined in the mrPRIinstPi register, *i* = 1, …, *n* − 1. The hardware must ensure that every time the CPU executes a write operation in these dynamic priority registers of nMPRA instances, the TTIID is updated (this can also be double buffered). This is possible because the value of the new priority must be communicated to the writing function, and the register number assigned to the instance for setting the priority is found in the register encoding or in the memory address used, depending on the deployment.

The scheduler type is selected only at the instP0 level of nMPRA, whose structure is found in Table 5. The structure of the priority selection register of an nMPRA instance is presented in Table 6.

cr0CPUID: Register with information about CPU resources. This register is wired to RESET.

mrPRIinstPi (dynamic priority register): Register for dynamic priority. It does not exist for instP0. After resetting, all bits are 0L (static priority is active). It is only accessible by instP0 or a dynamic hardware scheduler.

The dynamic priority scheduler must be implemented at the level of instP0 because it is the only instance that can stop all other instPi, which can write in the PRIinstPi register, which has access to the mechanisms for generating time, deadline, and WDT events of all nMPRA instances, as well as other useful functions. Enabling the dynamic priority scheduler can also be used to resolve issues related to priority inversion (i.e., priority ceiling protocol) or change the priority depending on the device status (normal, test, and service), among other functions. Usually, “low end embedded” does not provide special mechanisms for resource starvation, this being more specific to high-performance multi-user systems, i.e., those that work with groups of threads. However, because nMPRA_HW_RTOS has dynamic priorities and timers, for example, the aging scheduling technique can be used. Previous implementations have often used static scheduling, possibly by using the static scheduling algorithm RM. In [24], a specific implementation of a double priority algorithm is presented, whose purpose is to ensure that the RTOS remains functional, even if there is a transition from normal to another state (diagnosis, service, and test) during its operation. This change can lead to situations in which some tasks are delayed for an unacceptably long time, with possible unintended consequences. The algorithm ensures that each task is executed, even for those states that are different from the normal state, by changing a task with a priority that exceeds the amount of T-time in the normal state and placing it in one of the queues iq (queue with hanging threads) or ltq (queue with long threads) based on the length of the load execution. After the change disappears, the algorithm reverts to normal time-triggered operation. In this study, the logical structure for the scheduler was re-evaluated and modified by using the TTIID table, which connects the scheduled priority for instPi to the correct static identifier of that instance (see also Figure 6 and Figure 7).

### 4.3. Module for the Hardware Management of Mutexes

**Figure 8:**
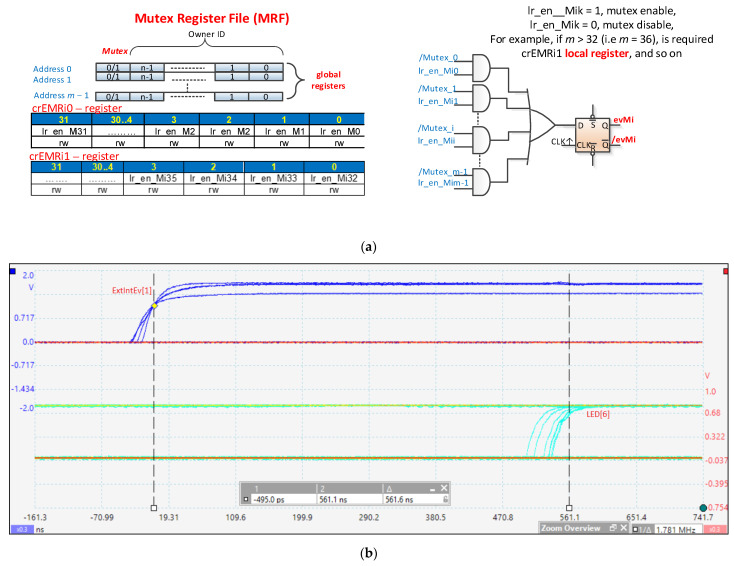
Hardware management of mutexes. (**a**) Implementation of mutex logic; (**b**) kernel latency based on the hardware search in EMRi registers (WCET = 561.6 ns, including the external interrupt threating, context switch, mutex unblocking/ blocking, and LED[6] ON).

Mutual exclusion is an important aspect of access to shared resources. The scheme proposed in [6] is partly set out in Figure 8. Here, Figure 8a represents *m* registers for *m* mutexes, which together form the mutex register file (MRF). A register contains the value of the mutex (0 = mutex free, 1L = mutex occupied) on the first bit and the static identifier of the owner (nMPRA instance number) on the next bits ($\left\lceil \log_{2}n\right\rceil$). These registers can be accessed by any instance of nMPRA, so it is a resource shared by all instPi. At the level of each instance, there is a scheme similar to the one illustrated in Figure 8a that allows the generation of an evMi event (see Figure 4) whenever an expected mutex is free. Each instPi of nMPRA can decide which mutex to consider using lr_en_Mi0, …, lr_en_Mim − 1 signals. These signals are stored in local registers called enable mutex registers (EMRi). There may be one or more EMRi registers depending on the number of mutexes implemented in the MRF. To synchronize with the processor clock, the type D flip-flop that stores the information on the positive edge of the CPU_clock is used. The attempt to write 1L on the mutex (the static identifier of the instPi on which the thread is running is automatically written) is successful if, after the CTC2 (copy control word to coprocessor 2—MIPS32) instruction is executed, 1L followed by the static identifier of the instPi to which the thread has written remains in the register used for writing the mutex bit. Otherwise, the mutex is busy. The mutex register can be read with a CFC2 (copy control word from coprocessor 2). If the mutex is busy, it can remain in the loop and wait for the mutex to be released. This solution is not recommended because it blocks the execution of lower (numerical greater) priority threads, and it reaches starvation if the mutex has been blocked by a thread with a lower priority than the running instPi (priority inversion). It is preferable to enter the blocked state by activating the lr_en_Mij signal (it is thread *i* that runs on instPi and mutex *j*), validating lr_enMi in the crTRi register and then running the generic instruction wait Rj, for example CTC2 Rj, crTRi for COP2 MIPS32 (Rj if it is a MIPS GPR). Upon entering the program sequence to handle this event, it is recommended to first delete the lr_en_Mij bit from the EMR (because lr_en_Mij is used to determine the highest priority instance that wants to take over the mutex). Afterwards, it is possible to try again to take the desired mutex.

The EMR registers are local and are found at the level of each instPi. After resetting the Mutex bit (MRFi) corresponding to the released mutex, the instPi_ID that executes the CTC2 instruction updates the mutex register automatically. After execution, in the register (indicated in the CTC2 instruction), the Mutex bit will be 0L, followed by the instance identifier that is executing the instruction, if the MRF and the running instance identifiers coincide. The Mutex can be released only by the owner. A major design problem is that an instance can be stopped at any time by a higher priority instPi that is ready to run. Both instances can be in a state that allows them to access the mutex at the same time. Moreover, there may still be a higher priority instance accessing the same mutex, for example. The mutex applies to the higher priority nMPRA instance, so a queue is created in which instances are ordered by priority. Recall that thread *i* is running on instPi. The nHSE_inhibit_CC nHSE signal can inhibit thread context switching in certain critical situations, as is the case with atomic accesses. For example, being accessed by all instPi, the mutex lock and release operations must be performed indivisibly with the help of nHSE_inhibit_CC internal nHSE signal. Depending on the number of mutexes, the HW_nMPRA_RTOS can have one or more EMR registers at the level of each instPi, as shown in Figure 8a.

Figure 8b presents the kernel latency for mutex implementation (Δ*t* = 561.6 ns) obtained by using the PicoScope 6404B oscilloscope by Pico Technology (St Neots, UK). The nHSE logic for mutexes was tested when the evMi (Table 2) event validated by the lr_enMi bit (Table 1) was set.

The hardware parallel search based on combination logic in Figure 8a enables the activation of the event evM0 (Figure 6) attached to instP0, thus generating the selection signal en_pipe_instP0 and the resources in the module HW_thread_0. The benchmark code to evaluate the synchronization mechanism is presented in [7]. The mutex implementation scheme automates the processes of blocking, waiting, and unblocking. In addition, a signal can be created in the system that indicates that all mutexes are occupied (busy_Mutexes). An important implementation aspect is that the occurrence of the evMi event does not guarantee access to the mutex because it may be required by a higher priority thread. The number of mutexes is determined using more complex criteria related to the specifics of applications, technological restrictions, the number of instPi, etc.

### 4.4. Hardware Module for Synchronization and Inter-Thread Communications

**Figure 9:**
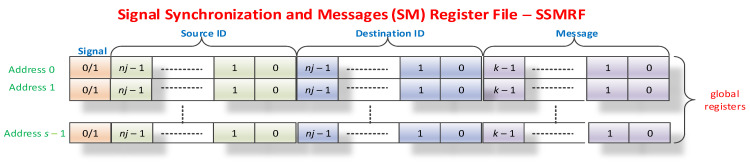
The synchronization and message register file.

Another important aspect of any RTOS is synchronization and communication between threads. For implementation, as for mutexes, defining a set of global registers with rapid access is proposed. These *s* registers with 2*nj* + *k* bits (Figure 9), which form the SSMRF (Signal Synchronization and Message Register File, originally named Event Register File in [6]), store the actual signal on the most significant bit; the static identifier (sIDm) of the source thread (which coincides with that of the instPi) that activated the event on the following *nj* $=\lceil \log_{2}n\rceil$ bits; the static identifier of the destination thread (dIDm) for which the event is sent on the next *nj* bits; and a message whose meaning remains at the discretion of the application programmer (it is possible that the field is not used) on the last *k* bits. The SSMRF behaves in one way when activating an event (Signal) and another way when the destination thread reads the event to find out who sent it and what message it sent. When a thread wants to activate a signal, a 32-bit value with the Signal bit 1L (Figure 9) is written on the grSSMR0 register and will receive a 1-bit signal value from the grSSMR0 register or a 0-bit signal value that represents a failure (most likely indicating no free SSMR). The hardware must perform a parallel search, if possible, in a single processor cycle, and the operation must be indivisible for the hardware scheduler. At this point, the register whose address has been returned is reserved, and when the instruction described above is executed, it writes the static identifier of the instPi on which the thread containing the instruction was executed on the source identifier field. Thus, it will be written on address *i*, obtaining the desired value from the grSSMRi register according to the structure in Table 7. Note that address 0 will never be returned as a grSSMRi register address. Thus, we have *n* = 4 for 16 instPi instances, and as a result, we have nine bits of control and identification and 23 bits for message identification, or they may even constitute a value (for example, a 23-bit integer, 21 bits if *n* = 5, or 27 bits if *n* = 2).

**Figure 10:**
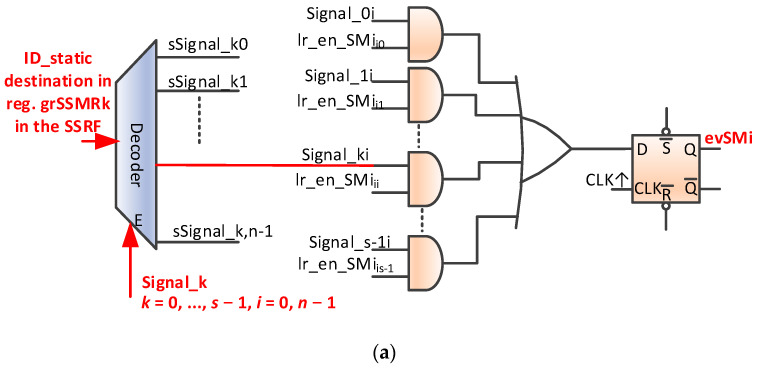

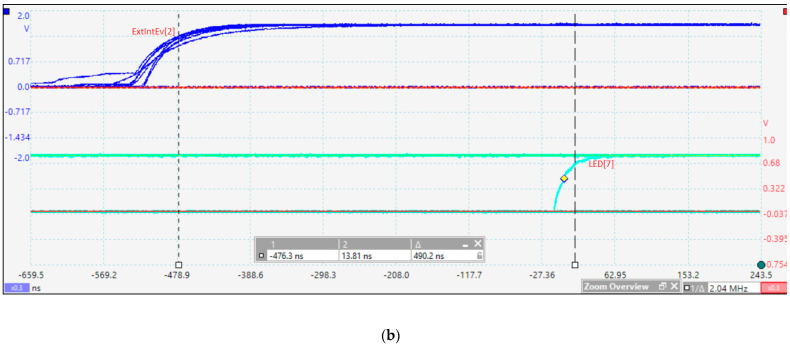
Hardware management of inter-thread communication mechanisms. (**a**) evSMi event generation logic; (**b**) inter-thread communication jitter (WCET = 490.2 ns, including the external interrupt threating).

The mechanism for validating SM-type events is analogous to that of mutex validation. Thus, at the level of each instance of nMPRA, there are one or more local registers crESSMrij (crESSMRi0, crESSMRI1, etc.; see Figure 9). Validation signals for instPi are called lr_en_SMjk (1L: the signal validates the generation of the evSMi event; 0L: validation is disabled for this signal). Similarly, the generation of the evSMi event at the level of instPi can use the same scheme as in Figure 8b, but the name is changed to *Mutexi* with Signali, lr_en_Mij with lr_en_SMij, and evMi with evSMi. These changes are presented in Figure 10a. When the destination task receives the evSMj event, it reads the SSMR0 register, and if the signal bit is 1L, the last bits contain the address of the first register that has the static identifier of the instPi on which the instruction is running in the destination field. Otherwise, no message is received (error). Reading must be continued until the thread no longer receives a message. Reading at dIDm destination can clear or not the signal bit (using the static identifier of the destination instPj instance). A possible hardware scheme for the mechanisms described above is shown in [6]. Signals can be used to implement more complex mechanisms that can also enable information communication through either pointers to specific message structures or the numeric value in the message field. The actual implementation is determined by the designer. Similarly, the number of SMs in the system depends on the designer, the specifics of the applications, technological restrictions, the number of instPi, etc. No significant improvements have been made in relation to the mechanisms described in [6].

In this study, we completed the scheme by adding a static identifier decoder destination for the message to each SSMRk register, which is validated by the appearance of a signal that can be picked up by its intended message signaling scheme for, in this case, instPi. Mutex and SM are very powerful mechanisms with very low run times and specific hardware support, which eliminates sequential searches in lists by using a parallel hardware search and reading the message in a thread without searching through software. Thus, all access is protected by indivisibility by eliminating the race condition and ensuring a single access time for all items in the list (see SSMRF registers). The kernel latency to an event can be two to four machine cycles if a higher priority event is not being executed.

Figure 10b illustrates the handling of the lr_enSMi event in the crTRi register shown in Table 1. The period of time illustrated in the oscilloscope capture (Δ*t* = 490.2 ns) includes handling an external interrupt event associated with instP2 (2.18 clock cycles), sending the message to instP0 (five cycles for the pipeline stages are necessary for the execution of the store word (sw) MIPS32 instruction), parallel searching in the hardware, and setting the flip-flop evSMi presented in Figure 10a (one cycle), setting the instP0_ready line in Figure 4b (one cycle), changing the context based on the selection of HW_thread_i related to instP0 (one cycle), jumping to the appropriate event handler (one cycle), and executing the load word (lw) instruction so that the LED[7] digital output is mapped in the data memory address space (five cycles).

### 4.5. The Module for Interrupt-Type Events

**Figure 11:**
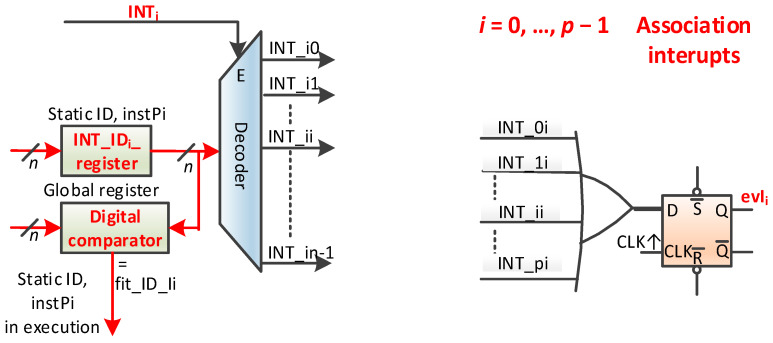
The programmable association register of an interrupt event with instPi (default with thread *i*) and the event generation scheme for that thread.

There are only exceptions in HW_nMPRA_RTOS. Interrupts are handled as interrupt events that do not require saving information because the thread resources are multiplied at each instPi level. Moreover, these interrupt events can be attached to and have priority to any thread (classically, interrupts are the highest priority sections of code that may or may not interact with the thread through RTOS API functions). This uniformizes the space of priority threads—interrupts. Otherwise, a priority inversion situation may occur here as well. An interrupt that sends a signal to a task with a certain priority can interrupt tasks that are higher in priority than that task. The proposed scheme for this module is presented in Figure 11. We note that an interrupt event can be associated with any instPi and thus with any thread *i*. This event inherits the instPi priority. We assume that there are *p* events of the interrupt type in the system. For each interrupt in the system, there is a global register with *n* useful bits, i.e., INT_IDi_register, that stores the static identifier of the task that the interrupt is associated with. Enabling the INTi (Figure 11) interrupt event validates the decoder, which, in turn, activates one of the INT_i0, …, INT_in − 1 signals.

**Figure 12:**
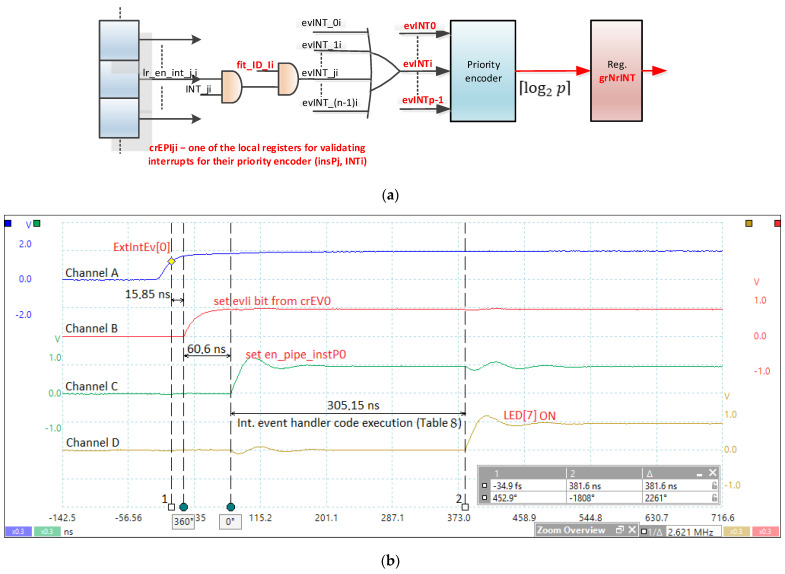
Hardware management of interrupt events. (**a**) The logical schema of generating the number associated with the interrupt event that is also its priority; (**b**) Interrupt event latency (Δ*t* = 381.6 ns): asynchronous external signal triggering = 15.85 ns (Channel A), kernel latency = 60.6 ns (Channel B–Channel C), and real-time response = 305.15 ns—en_pipe_instP0 = 1, CPU executes the first C instruction of the handler routine and set LED[7] (Channel D).

The OR logical gate in Figure 11 can collect all interrupts in the system if, for example, all are attached to instPi and if all *p* registers INT_IDi_register (*i* = 0, …, *p −* 1) have the value *i*. D flip-flop is intended to synchronize a random occurrence of an INTi interrupt event by creating the event. The schema also produces the fit_ID_Ii signal that can activate the interrupt attached to instPi (threads) at the input to the priority encoder (see also Figure 12a). This schema has some powerful and interesting features:

- There must be no specialized controller for interrupts;
- Interrupt events inherit the thread priority, i.e., instPi;
- A thread can attach none, one, more, or even all of the *p* interrupts in the system;
- For interrupt events attached to the same instPi, the priority is set by the programmer or a hardware schema;
- Interrupts attached to instPi can preempt a lower priority thread, but they cannot interrupt the execution of the thread to which it is attached or a higher priority thread;
- An interrupt event can be attached to a single thread;
- The interrupt-type event handler can be a thread;
- All interrupts can be attached to a single instPi;
- The interrupt does not reset the pipeline of other instPi;
- This does not involve saving and restoring thread contexts;
- Interrupts can be nested;
- Interrupt priorities can be dynamic (by reattaching to instPi or by changing the priority of the instPi to which it is attached).

The proposed scheme is very versatile and can implement multiple models of work with interrupts in a real-time execution. In terms of prioritization, a schema deficiency occurs when multiple interrupt events are attached to instPi; a loop test is performed in which prioritization is implemented through software and the response time depends on the loop position. Based on the ideas expressed in [22] and [23], a possible improvement is presented in Figure 12a. The priorities of interrupts, if they are all attached to a single instPi, is INT0 for the highest priority and INTp − 1 for the lowest priority. From this point of view, the priorities are fixed. To take into account only the interrupts of instPi (the highest priority) at a time, local crEPIji registers are provided (*j* is instPj, and *i* is the number of the register attached to interrupt *i*). This register contains one bit for each of the *p* interrupts. If the bit is 1L, then the interrupt is attached to the thread, and the INT_IDi register must be written with the identifier of that thread. This correspondence must be provided for any interrupt attached to instPi. If this connection is not performed for an interrupt, then it will never generate an event because the evINT_ji signal will be 0L. The evINT_ji signal is activated if the instPj to which the event was attached is running, with the fit_ID_i active signal being 1L. The evINTi signal, for example, handles all possible attachments of the interrupt to instPi, but it is mandatory that an interrupt be attached to a single thread. Therefore, because only one instPj is active at any given time, only interrupts of that instance can be active when entering the priority encoder. Thus, using the grNrINT register, the *j* instance will read the interrupt number with the highest priority. A bit will signal a possible error in the sense that there is no active interrupt. Attaching an interrupt event to a specific instPi (equivalent to attaching the event to thread *i*) is similar to setting a priority level for an NVIC-handled interrupt in ARM processors. The difference is that, in this case, the priority of a thread acting as an interrupt event handler can increase.

Figure 12b illustrates the practical measurements of the interrupt event latency based on the scheduler implemented in HW_nMPRA_RTOS. In this test, the interrupt event was validated by means of the lr_enIi bit in the crTRi register (with the highest priority). The time period shown in the oscilloscope capture (Δ*t* = 381.6 ns) includes external signal triggering, instPi_ready flip-flop (Figure 4a), setting the en_pipe_instP0 signal for instP0 (Figure 6), jumping to the appropriate handler (Table 8), executing the sw MIPS32 instruction, and changing the digital output state mapped in the address space of the data memory.

Figure 12b illustrates the time period from the occurrence of the external ExtIntEv[0] signal, corresponding to the pressing of a button in the development kit, until its capture in the interrupt event bit (evIi) from crEVi register. This jitter is 15.85 ns, which depends on when the trigger appears relative to the positive edge of the input signal (Channel A) to the next positive edge of the CPU_clock (Figure 5a). Because the CPU clock period is 30.3 ns, the jitter can range from 0 to 30.3 ns.

The evaluation of the performance showed that, despite the additional hardware costs, the implementation of the scheme in Figure 12a is mandatory for minimize the kernel latency to external stimuli by the immediate execution of the handler corresponding to the treatment of interrupt events (IEH). The schema can be expanded to cover all events, as shown in Table 9.

### 4.6. Module for Generating Time Events and Counters for the Running Times of instPi

**Figure 13:**
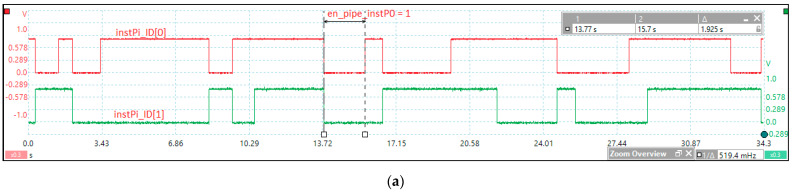

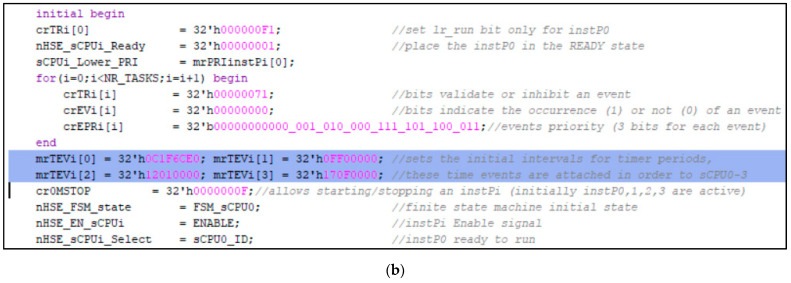
Time events in the nMPRA. (**a**) Oscilloscope signals for testing instPi_ID in the RUN state; (**b**) the initialization section of the hardware scheduler in Verilog HDL using Vivado development tools.

This module generates time events at the level of each instPi: deadline 1 (D1i), deadline 2 (D2i), and watchdog timer (WDTi) tick event types. Two counters are provided to measure the operating cycles (CNTExi) and the stationary ones (CNTIdlei) at the level of each instPi and at the level of the CPU (CNTEx and CNTIdle). Figure 13a presents tick events with the signals instPi_ID[1:0] modified based on the signals lr_enTi and evTi in Figure 4a and the current instPi_ID in Figure 6. Figure 13b shows part of the initialization section of the nHSE registers, i.e., setting periods for tick events (mrTEVi[3:0]).

### 4.7. Other Modules Implemented in the nHSE

This section describes additional modules implemented in the test architectures that are not mentioned above.

The memory protection (MP) module includes *x* sets of three registers that store the basic address of the memory mode, its end address, and the status and control of the mode (invisible and cannot be accessed, read only, write only, read/write, execute only, read/execute, write/execute, static identifier (instPi_ID) of instPi, etc.) with the generation of memFault interrupts or a break event.

Each set can be attached to any instance and any memory space. Usually, there are as many sets as there are instPi, but a set can be attached to any nMPRA hardware instance.

The Debug module provides breakpoints for data addresses/instructions (for reading and writing execution), for events (including taking a mutex or signal, no free mutex, no free signal), and for instPi activation. At a breakpoint, the program that runs on that instance stops running, or the nMPRA clock is blocked, after which the program can run step by step. This module can also determine the status of the processor (all registers available to the programmer and special registers) and change the status of nMPRA.

## 5. Discussion

The further development of this project will enable rapid response times to events and controllable deterministic behavior for both simulations and practical implementation using FPGA technology. The proposed research project is a novel contribution, and similar implementations are not described in the specialized literature. In this paper, previous projects and implementations that are comparable to the proposed architecture are presented and analyzed. Table 10 presents a comparison between the nMPRA architecture and a few other performant processor implementations. The proposed HW_nMPRA_RTOS system with a real-time hardware scheduler is a novel design. The JTAG debugging module was used for the testing and verification of the proposed processor concept. In the design and testing stages, we used the Vivado integrated simulator, Verilog testbench, ChipScope ILA, and PicoScope 6404B.

The initialization and operation of internal scheduler registers and hardware logic were validated using assembler program code benchmarks. For the project validation and verification methodology, the proposed system was compared with other architectures based on tests performed by the authors, in which each implementation was synthesized and implemented in the FPGA.

By parameterizing the Verilog HDL code, it is possible to analyze the CPU working frequency and FPGA resources needed for CPU extensions. The Virtex-7 resource requirements for implementing the HW_nMPRA_RTOS architecture in different configurations and for comparing performances with other projects are presented. The project validation analyzes many aspects of the system: the bus structure, the hardware resources, and the data coherency. This paper proposes a multitasking RTS architecture. We focus on hardware solutions, but we also analyze software issues of time-predictable RTSs. The basic building block of the system is the processor core based on the idea of thread pipelining. Moreover, the pipeline structure of a core based on nMPRA+nHSE and MIPS32 is flexible and can be configured for a given instPi. Table 11 presents the resource requirements for implementing different CPU architectures.

**Figure 14:**
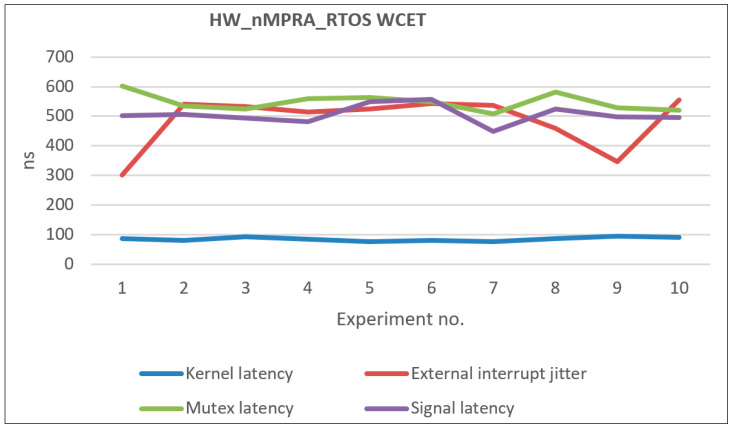
The distribution of the WCETs based on HW_nMPRA_RTOS relative to the events associated with instP0.

Table 12 presents the WCET coefficients in microseconds following the processor performance evaluation presented in this paper. Figure 14 illustrates the distribution of the obtained WCETs based on multiple measurements, the data being relative to the events associated with the highest priority instPi. The following times have been measured: the relative time for the initialization and boot operation of the HW_nMPRA_RTOS, the time required for the task context switching operation in the least favorable case when the assembly line contains atomic instructions, and the task activation time by an interrupt event.

The following aspects contributed to the WCET coefficients obtained and presented in Table 12: the multiplication of resources for each instPi (HW_thread_i), the hardware implementation of the nHSE scheduler [28] at the level of COP2, and designing the synchronization and communication mechanisms as part of the nHSE module [7].

The main technical objective of our project is to implement the HW_nMPRA_RTOS concept for a time-predictable embedded system. The dynamic scheduler has a major effect on the RTOS overhead. In the future, we are considering the hardware implementation of an EDF scheduling algorithm and a priority ceiling protocol defined by OSEK. For BASELINE, we will use BL_RISC-V with four-stage pipeline.

## 6. nMPRA Programming Paradigms

**Figure 15:**
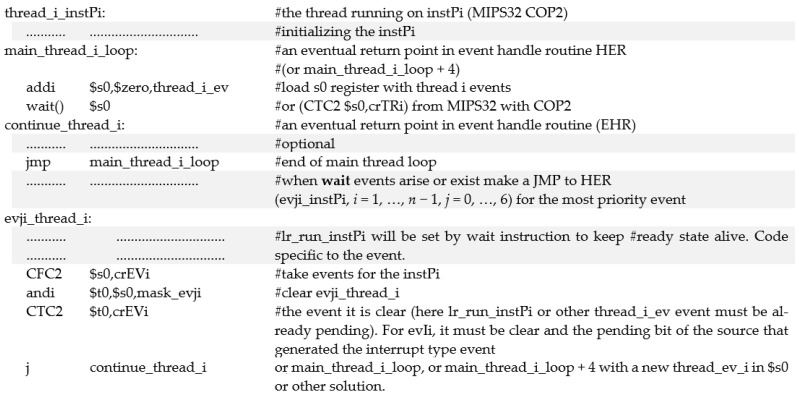
A minimal template for thread *i*.

With the mechanisms implemented in HW_nMPRA_RTOS, programmers can write RTOS applications in a simple manner without switching contexts for multiple register saves or managing complex data structures; without calling RTOS functions; without message queues; without a complex time management function, usually with a variable duration depending on the application context; without managing queues with the thread status by the scheduler; and without a controlled software dispatch. These operations can only be performed by simple reading/writing operations in HW_nMPRA_RTOS registers. A general example is shown in Figure 15, where the initialization sequence initiates its thread and hardware resources (HW_thread_i) through an application. If necessary, lr_en_Xi-type bits can be validated for events to be used by thread_i on the instPi instance.

When the wait instruction is called (when lr_run_instPi = 1L), it compares events validated with thread_i_ev with events already pending. If one or more events in thread_i_ev are already pending, then wait loads the PC with the start address of the event handler routine (EHR) of the highest priority event (if there are more). If no events are waiting, then the wait Rj instruction deletes lr_run_instPi = 0L, the thread exits the ready-to-run state, and through the scheduler, HW_nMPRA_RTOS dispatches another instPi (the highest priority that is ready to run) to run the associated thread. When an event occurs for thread *i* (it becomes ready to run) and when instPi has the highest priority, the execution of the wait Rj instruction that sets the lr_run_instPi bit resumes and transfers control to the EHR corresponding to the highest priority event. In the EHR, the bit from the crEVi register must be deleted (with lr_run_instPi = 1L), and the time depends on the frequency of the occurrence and the behavior of the event. It is usually cleared at the end of the EHR. If the event is an interrupt, then the source that generated it must also be cleared. With the CTC2, CFC2, MFC2 (move word from coprocessor 2), and MTC2 (move word to coprocessor 2) instructions, all HW_nMPRA_RTOS functionalities are easily managed. 

In the future, we will propose for these functions similar RTOS names often used at this level (Keil RTX5, uC_OS III—Micrium, FreeRTOS, etc.). Several applications will be tested and additional hardware will be added for other specific RTOS facilities. 

## 7. Conclusions

The proposed architecture is based on predictive execution using the nMPRA concept to satisfy the timing constraints required in real-time applications without exceeding the imposed limit of power consumption. Moreover, the simulation, synthetization, and implementation of this project in an FPGA will enable the development and debugging of the appropriate applications. The research carried out for this paper was completed by a series of practical tests, and the scientific results were validated based on well-chosen experiments.

In this context, the nMPRA + nMSE architecture will be implementable in silicon, scalable, and configurable. HW_nMPRA_RTOS is an architectural concept designed for high performance and energy-efficient implementation by combining the MIPS32 or RISC-V architecture, ISA extensions, explicit management of the memory hierarchy, and compiler support to meet performance requirements for RTSs used in industry and beyond. The FPGA implementation can meet a wide range of performance requirements by scaling the number of instPi, configuring the events at the level of each HW_thread_i, or adding specific extensions. The economic benefits of the solution include the significant growth of productivity due to its easy integration into software applications of new RTSs, even in the IoT sector. The proposed custom CPU implementation can provide static or dynamic priorities for interrupts, depending on the priority of the instPi to which they are attached. This solution may be the basis of a variety of specific applications for the monitoring and control of industrial processes. In this innovative project, the feasibility and performance of the HW_nMPRA_RTOS implementation were tested using a Virtex-7 FPGA circuit. The experimental results obtained by implementation in a physical chip can lead to major improvements in the development of real-time applications. The ability to flexibly set processor instance priorities and dynamically attach interrupt events to any instPi provides, in addition to quick responses to events, increased performance for RTS applications.

## 8. Patents

The nMPRA and nHSE concept presented in this paper is patented in Germany, Munich (DE202012104250U1, June 2012).

## Figures and Tables

**Table 1:** The meaning and location of bits for the crTRi register.

31	30, …, 8	7	6	5	4	3	2	1	0
0	0, …, 0	lr_run_instPi	lr_enSMi	lr_enMi	lr_enIi	lr_enD2i	lr_enD1i	lr_enWDi	lr_enTi
		rw	rw	rw	rw	rw	rw	rw	rw
Value after CPU RESET									
0	0, …, 0	1	0	0	0	0	0	0	0

lr_enTi = 1/0 Enable or disable the event generated by the timer (preferably a periodic event); lr_enWDi = 1/0 Enable or disable the event generated by the WDT; lr_enD1i = 1/0 Enable or disable the event generated by the critical limit (deadline) 1; lr_enD2i = 1/0 Enable or disable the event generated by the critical limit (deadline) 2; lr_enInti = 1/0 Enable or disable events caused by interrupts; lr_enMi = 1/0 Enable or disable events generated by mutexes; lr_enSMi = 1/0 Enable or disable events generated by signals and messages (synchronization and communication between tasks); lr_run_instPi = 1/0 Enable or disable program execution on instPi.

**Table 2:** The meaning of the bits for the crEVi control register.

31	30, …, 8	7	6	5	4	3	2	1	0
0	0, …, 0	lr_run_instPi	evSMi	evMi	evIi	evD2i	evD1i	enWDi	evTi
rw		rw	rw	rw	rw	rw	rw	rw	rw

evTi = 1/0 OR The event generated by the timer; evWDi = 1/0 OR The event generated by the WDT; evD1i = 1/0 OR The event generated by the deadline 1; evD2i = 1/0 OR The event generated by the deadline 2; evIi = 1/0 OR Interrupt type events; evMi = 1/0 OR Events generated by mutexes; evSMi = 1/0 OR Signal events; lr_run_instPi = 1/0 OR Copy of the homologous bit from crTRi.

**Table 3:** The significance of the bits for the cr0MSTOP register.

31	30, …, 4	3	2	1	0
mr_stop_instP31	mr_stop_instPi, *i* = 4, …, 30	mr_stop_instP3	mr_stop_instP2	mr_stop_instP1	–
rw	rw	rw	rw	rw	rw

mr_stop_instPi (*i* = 1, …, 31) = 1/0 instPi is validated or stopped.

**Table 4:** Truth table for the identifier encoder (i.e., priority) for eight nMPRA instances.

instP0_ ready	instP1_ ready	instP2_ ready	instP3_ ready	instP4_ ready	instP5_ ready	instP6_ ready	instP7_ ready	idPri3	idPri2	idPri1	idPri0
1	x	x	x	x	x	x	x	0	0	0	0
0	1	x	x	x	x	x	x	0	0	0	1
0	0	1	x	x	x	x	x	0	0	1	0
0	0	0	1	x	x	x	x	0	0	1	1
0	0	0	0	1	x	x	x	0	1	0	0
0	0	0	0	0	1	0	x	0	1	0	1
0	0	0	0	0	0	1	x	0	1	1	0
0	0	0	0	0	0	0	1	0	1	1	1
0	0	0	0	0	0	0	0	1	0	0	0

**Table 5:** Bit significance for the cr0CPUID register.

31	30, …, 4	3	2	1	0
CPUID31	CPUIDi, *i* = 4, …, 30	CPUID3	CPUID2	CPUID1	CPUID0
rw	rw	rw	rw	rw	rw

CPUID 4−0 Indicates the number of instPi; CPUID 6−5 Indicates the CPU version; CPUID 8−7 Indicates the dynamic scheduler version, 00 = static scheduler (sel_sched_dyn = PUID7 + PUID8, see also Figure 6), otherwise the dynamic scheduler is implemented through software or with an external FPGA. At RESET PUID7 = CPUID8 = 0; CPUID 9 = 1/0 It has or not the events priority encoder; CPUID 10 = 1/0 It has or not the interrupts priority encoder; CPUID 11 = 1/0 Has direct trap cells to the event handler.

**Table 6:** Bit meaning for the mrPRIinstPi register.

31	30, …, 4	4	3	2	1	0
		PriD4	PriD3	PriD2	PriD1	PriD0
0	0	rw	rw	rw	rw	rw

PriD4, …, 0 = 0 Priority is static; PriD4, …, 0 ≠ 0 Represents the dynamic priority of the task (between 1 and *n* − 1, where *n* is the number of instPi.

**Table 7:** The meaning of the bits for the grSSMRi register and the SSMRF.

	*nj* − 1		1	0	*nj* − 1		1	0	*k* − 1		1	0
0/1	sIDnj − 1		sID1	sID0	dIDnj − 1		dID1	dID0	messk − 1		mess1	mess0
Signal	Source Task Identifier				Destination Task Identifier				Message			
rw	rw	rw	rw	rw	rw	rw	rw	rw	rw	rw	rw	rw

Signal = 1/0 Signal with active or inactive message; *nj* = ⌈log_2 *n*⌉ sIDm—bits for static source identifier, dIDm—bits for destination static identifier.

**Table 8:** The interrupt event handler MIPS32 code for testing CPU data path and kernel latency.

Application Description	MIPS32 Code for Interrupt Event Latency Measurement
instP0 is executed for treating an interrupt event, with the highest priority of all activated events.	//instP0 interrupt event handler 200e0000, //addi (Add Immediate), SignExtImm = 0000 00000000, //nop (no operation) //The following two instructions write the value 32’h30000000 //in the GPR register r14, to address the LEDs 200e0003, //addi (Add Immediate), SignExtImm = 0003, rd = r14 000e7780, //sll (Shift Left Logical), Shamt = 30, rd = rs = r14 //for LEDs: MIPS32_Data_IO_Mem_Addr[29:26] = 4’b1100 200c00f0, //addi, SignExtImm = 00f0, rd = r12, write the value 32’h000000f0 in the r12 //register to switch ON the LEDs[7:4] (Virtex-7 Development Kit) 00000000, //nop (no operation) adcc0000, //sw (Store Word), save r12 COP0 to the address stored in r14 //r14contain address of I/O mapped in the memory data address space 00000000, //nop (no operation) 48c1ffff, //movcr, the wait instruction causes the next context switch based on nHSE

**Table 9:** The trap address for the IEH generated by the hardware.

no	Event	Thread 0 (instP0)	Thread *i* (instPi)	...	Thread *n* − 1 (instPn − 1)
	ADDRESS, start to 0				
0	Reset_i	(instP0_ID × 16 + no) × 4	(instPi_ID × 16 + no) × 4	...	(instPn − 1_ID × 16 + no) × 4
1	Reserved_1i	idem	idem	...	idem
2	NMI_i	idem	idem	...	idem
3	HardFault_i	idem	idem	...	idem
4	MemFault_i	idem	idem	...	idem
5	InstrFault_i	idem	idem	...	idem
6, …, 7	Reserved_6, …, 7i	idem	idem	...	idem
8	evTi	idem	idem	...	idem
9	evWDi	idem	idem	...	idem
10	evD1i	idem	idem	...	idem
11	evD2i	idem	idem	...	idem
12	evIIi	idem	idem	...	idem
13	evMi	idem	idem	...	idem
14	evSMi	idem	idem	...	idem
15	Reserved_15i	idem	idem	...	idem
		ADDRESS start to base_address_evI = (16 × n × 4)			
16	evI0	base_address_evI + ((No − 16) × 4) = base_address_evI			
16 + p − 1	evIp − 1	base_address_evI + ((No − 16) × 4) = base_address_evI + (p − 1) × 4			

Note: A global register can be added to enable the software to generate interrupt events.

**Table 10:** Comparison between different CPU architectures based on implementation and hardware support.

Project	Architecture Type	CPU Scheduler	Frequency	FPGA	Pipeline Stages/Power Consumption
nMPRA4	MIPS32	Static/Support for dynamic scheduler implemented in hardware	33 MHz	Xilinx Virtex-7, XC7VX485T-2ffg1761C	5 stages/0.432 W
uRV Core [16,21]	RISC-V (RV32IM ISA)	Software	100 MHz/94 MHz	Xilinx Spartan-6/Altera Cyclone-4 E	4 stages/0.737 W (Device Static)
nMPRA4 [25]	RISC-V	Static nHSE/hardware	100 MHz	Xilinx Virtex-7, XC7VX485T-ffg1761	3 stages
Cortex M3 [26]	ARM	Software	40 MHz	Xilinx Spartan-7, xa7s50csga324-2I	3 stages/0.255 W
biRISC-V [27]	RISC-V (RV32IMZicsr)	Software	>50 MHz	Xilinx Nexys4 DDR	Superscalar (dual-issue) in-order 6 or 7 stage pipeline

**Table 11:** The use of FPGA resources based on different CPU implementations.

Resources/SoC Project	XC7VX485T-2ffg1761C Virtex-7 Resources	uRV Core [16,21]	nMPRA (RISC-V) [25]	biRISC-V [27]	nMPRA (MIPS32) (4 instPi)	nMPRA (MIPS32) (16 instPi)
LUT	303,600	1271	39,882	11,326	16,014	58,774
LUTRAM	130,800	1	5725	1	814	946
FF	607,200	936	26,285	6678	8613	31,916
BRAM	1030	17	0.50	16	148	148
IO	700	9	89	327	32	32
BUFG	32	1	12	1	15	15

**Table 12:** WCET parameters related to the kernel functions based on HW_nMPRA_RTOS.

WCET Parameters	HW_nMPRA_RTOS WCET (33 MHz)
Thread context switch	0.033 µs (Figure 5)
Selection of the next thread to execute (scheduler time)	0.043 µs (Figure 5b)
Preempt a task instance (kernel latency)	0.060 µs
Treating an asynchronous external interrupt	0.381 µs (attached to instP0) (Figure 12b)
Executive booting and configuration	21.8 µs
Create/Stop instPi (cr0MSTOP)	0.151 µs
Activation and prioritization of a periodic task	0.242 µs
Read/write task state (1 parameter—32 bit)	0.165 µs
Enable/disable a mutex or semaphore	0.132 µs (mutex)
Lock/unlock a mutex or semaphore	0.212 µs (mutex)

## Data Availability

Data sharing not applicable.
